# Transabdominal intestinal ultrasound versus transmural histopathological findings in severe ulcerative colitis requiring colectomy

**DOI:** 10.1093/crocol/otag017

**Published:** 2026-03-07

**Authors:** Hiromu Morikubo, Jun Miyoshi, Akimasa Hayashi, Haruka Komatsu, Hiromi Yonezawa, Minoru Matsuura, Junji Shibahara, Tadakazu Hisamatsu

**Affiliations:** Department of Gastroenterology and Hepatology, Kyorin University School of Medicine, Tokyo, Japan; Department of Gastroenterology and Hepatology, Kyorin University School of Medicine, Tokyo, Japan; Department of Pathology, Kyorin University School of Medicine, Tokyo, Japan; Department of Gastroenterology and Hepatology, Kyorin University School of Medicine, Tokyo, Japan; Department of Gastroenterology and Hepatology, Kyorin University School of Medicine, Tokyo, Japan; Department of Gastroenterology and Hepatology, Kyorin University School of Medicine, Tokyo, Japan; Department of Pathology, Kyorin University School of Medicine, Tokyo, Japan; Department of Gastroenterology and Hepatology, Kyorin University School of Medicine, Tokyo, Japan

**Keywords:** colectomy, histopathology, intestinal ultrasound, transmural disease, ulcerative colitis

## Abstract

**Background:**

Transabdominal intestinal ultrasound (IUS) is a promising, non-invasive tool for monitoring ulcerative colitis (UC). This modality has the advantage of assessing intestinal inflammation transmurally, suggesting that UC can be considered a transmural disease. Determining what transabdominal IUS findings indicate in terms of histopathology would improve its value in assessing disease activity. However, associations between sonographic and histopathological findings have not yet been established for active UC. To address this gap, we investigated patients with active UC who underwent colectomy following IUS examination.

**Methods:**

Patients who underwent total colectomy for severe active UC within 1 week of undergoing transabdominal IUS at our facility between December 2020 and March 2023 were consecutively included in this study. Sonographic and histopathological findings were compared for each colonic segment in these patients.

**Results:**

Four patients underwent IUS 3-6 days before colectomy, which was performed due to insufficient response to medical treatment. IUS findings, particularly loss of bowel stratification and increased color Doppler signals, were associated with severe inflammation and vascular proliferation in the transmural colon, including the subserosa. Thickened muscularis propria was also observed in inflamed colonic segments; this may have contributed to the increased bowel wall thickness according to IUS.

**Conclusions:**

This is the first report comparing IUS findings and transmural pathological features in active UC. It provides an imaging atlas and clinical insights into the role of IUS in determining transmural histopathological inflammatory status in patients with active UC.

## Introduction

Ulcerative colitis (UC), a chronic inflammatory bowel disease (IBD) of unknown etiology, is characterized by repeated relapses and remissions.^1^ Advances in treatment options and therapeutic strategies have enabled more patients to achieve remission and better long-term prognoses.^2^ However, some patients respond poorly to multiple molecularly targeted therapies and require surgery.^3^ The concept of the treat-to-target strategy is now widely accepted for optimizing treatment for UC. Monitoring disease activity is key to When using this strategy.^4^ The current gold standard for assessing of UC activity is colonoscopy (CS), findings being expressed, for example, as the Mayo endoscopic subscore (MES).^1^ Meanwhile, transabdominal intestinal ultrasonography (IUS) has recently been considered a promising practical tool for monitoring UC because this modality is non-invasive and can be repeated. Additionally, sonographic findings and scores can be used to estimate the MES^5^^,^^6^ as well as to predict responses of patients with UC to treatment.^7^

While mucosal assessment has traditionally been considered critical in evaluating inflammation in patients with UC, a recent report has suggested that UC can be regarded as a transmucosal disease, implying that examining the entire bowel wall in patients with UC would provide useful information.^8^ Relevant to this, IUS has the advantage of enabling real-time, non-invasive transmural assessment of colonic inflammation. Meanwhile, determining what transmural sonographic findings denote in terms of histopathology findings remains a crucial challenge in establishing IUS as a means of evaluating the transmural inflammatory status in patients with UC. In some studies, biopsy specimens have been used to compare these patients’ IUS and histopathology findings.^9^ However, endoscopic biopsy specimens include mainly mucosa and some submucosa and therefore do not enable evaluation of all layers of the colon wall. Comparing transabdominal IUS images and transmural histopathology is currently an appropriate means of assessing the inflammatory activity of UC in daily practice. Some studies have compared histopathological findings in surgical specimens and sonographic findings, particularly using postoperative water-immersion ultrasound, to clarify normal colon wall structure.^10^ However, resected specimens are no longer functional and do not enable assessment of the vascularity of the inflamed colon wall.

To address these challenges, this is the first report to investigate the transabdominal IUS findings and their correlation with transmural histopathological features in patients with acute severe UC who underwent IUS prior to colectomy.

## Methods

### Study subjects and design

Patients who underwent colectomy for severe active UC within 1 week of taking transabdominal IUS examination from December 2020 to March 2023 at our Hospital were successively included in this study. UC was diagnosed based on the Inflammatory Bowel Disease Guidelines of the Japanese Society of Gastroenterology.^1^

### Sonographic and histological assessment

Transabdominal IUS findings and histopathological findings in each colonic segment (ascending colon [A/C], transverse colon [T/C], descending colon [D/C], and sigmoid colon [S/C]) were compared within individual patients. IUS findings, including major sonographic parameters such as bowel wall thickness (BWT; normal BWT was defined as < 3 mm),^11^ bowel wall stratification (BWS), bowel wall vascularity assessed by modified Limberg score (mLS), and inflammatory mesenteric fat (i-fat),^11^ were evaluated by two gastroenterologists (H.M. and J.M.) certified by the International Bowel Ultrasound (IBUS) Group for IBD-IUS. Histopathological findings of the total colectomy specimens were assessed by a board-certified pathologist (A. H.). Although the pathologist was aware that these specimens were from UC colectomies, the pathologist was blinded to the IUS images and all other clinical data. The severity of inflammation was determined based on the pathologist’s diagnostic report, in which inflammation is described in 3 categories (mild, moderate, severe), and all cases were subsequently re-evaluated by the same pathologist. The vascular proliferation was evaluated by the pathologist at low magnification (objective lens 4×) based on the number of visible vessels. The majority of these were venous structures, although small arterioles could occasionally be included. Capillaries were not counted, and intimal hypertrophy was not considered. When approximately twice as many vessels were observed at low magnification compared with the normal counterpart, the finding was defined as vascular proliferation.

## Results

Three patients with ulcerative pancolitis and one patient with left-sided UC who had undergone colectomy were analyzed in this study (Table 1). All four patients had severe disease activity that was refractory to molecular-targeted medication. IUS was performed 3-6 days before colectomy. Each patient’s sonographic and pathological findings are summarized in Table 2.

**Table 1:** Clinical, biochemical, endoscopic, and ultrasonographic characteristics.

Patient No.	1	2	3	4
**Age (years)**	68	16	78	29
**Sex**	Male	Female	Female	Male
**Age of onset (years)**	67	16	70	28
**Extent of disease**	Left-sided	Pancolitis	Pancolitis	Pancolitis
**Prior therapies up to surgery**	5-ASA, VDZ	5-ASA, PSL, GMA, ADA, IFX, CyA, VDZ	5-ASA, IFX	5-ASA, PSL, IM, IFX
**Interval between IUS and surgery (days)**	5	3	3	6
**Hb (g/dL)**	11.4	8.7	11.1	8.8
**Alb (g/dL)**	2.3	1.8	1.7	2.2
**CRP (mg/dL)**	27.6	1.13	23.39	5.63
**MES**	3	3	N/A	3

Abbreviations: 5-ASA = 5-aminosalicylic acid; ADA = adalimumab; Alb = albumin; CRP = C-reactive protein; GMA = granulocyte and monocyte adsorption; Hb = hemoglobin; IFX = infliximab; IM = immunomodulator; IUS = intestinal ultrasound; MES = Mayo endoscopic subscore; PSL = prednisolone; VDZ = vedolizumab.

**Table 2:** Comparison between intestinal ultrasound and histopathological findings.

		Sonographic findings					Histopathological findings						
Patient No.	Location	BWT (mm)	Thickened BWT	BWS	mLS	i-fat	Inflammation	Vascular proliferation	Edema	Thickened muscularis propria	Fibrosis in submucosal layer	Vascular proliferation in the subserosa	fat proliferation in the subserosa
**1**	A	1.2	–	Preserved	0	–	–	–	–	–	Mild	–	–
	T	0.9	–	Preserved	0	–	–	–	–	+	+	–	–
	D	6.3	+	Unclear	4	+	Severe	+	–	+	–	Focal	Mild
	S	3.1	+	Loss	3	+	Severe	+	–	+	–	+	Mild
**2**	A	5.7	+	Loss	3	+	Severe	+	–	+	+	+	–
	T	5.7	+	Loss	4	+	Severe	+	+	+	Mild	–	–
	D	6.6	+	Loss	4	+	Severe	+	+	+	Mild	+	–
	S	5.0	+	Unclear	4	+	Severe	+	+	+	+	–	–
**3**	A	5.2	+	Loss	4	+	Moderate to severe	+	+	–	+	Focal	+
	T	9.1	+	Loss	4	+	Severe	+	+	+	–	–	–
	D	5.6	+	Loss	4	+	Severe	+	–	+	–	+	+
	S	5.9	+	Loss	3	+	Severe	+	+	+	+	+	–
**4**	A	9.9	+	Unclear	3	+	Severe	+	+	+	–	–	+
	T	6.9	+	Unclear	3	+	Moderate to severe	+	–	+	–	+	–
	D	6.5	+	Unclear	3	+	Severe	+	–	+	–	+	–
	S	4.9	+	Loss	3	+	Severe	+	–	+	–	+	–

Abbreviations: A = ascending colon; BWS = bowel wall stratification; BWT = bowel wall thickness; D = descending colon; IUS = intestinal ultrasound; mLS = modified Limberg score; S = sigmoid colon; T = transverse colon.

### Case 1

**Figure 1:**
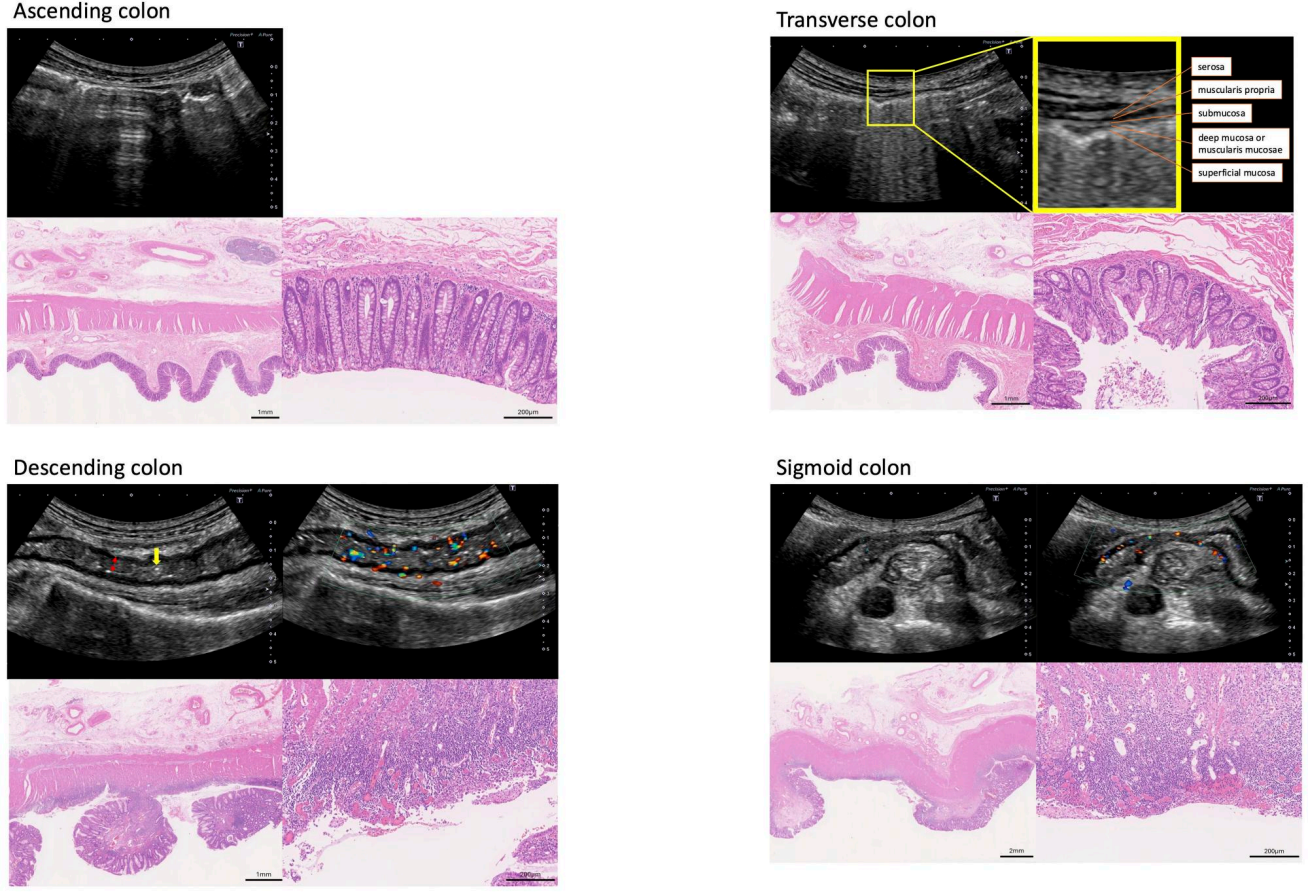
Transabdominal intestinal ultrasound and histopathological findings in Case 1. Sonographic findings: Ascending colon and transverse colon: bowel wall thickness within normal limits and bowel wall stratification preserved. The normal bowel stratification was observed, particularly in the transverse colon (enlarged view). Descending colon: thickened bowel wall, unclear stratification (double arrow), modified Limberg score 4, ulcers (+) (single arrow), i-fat (+). Sigmoid colon: thickened bowel wall, loss of stratification, modified Limberg score 3, ulcers (+) (single arrow), i-fat (+). Histopathological findings (H&E staining): Ascending colon: mild fibrosis in the submucosal layer. Transverse colon: fibrosis in the submucosal layer and thickened muscularis propria. Descending colon: deep ulceration, severe inflammation with surface inflammatory granulation tissue with vascular proliferation, thickened muscularis propria, focal vascular proliferation, and mild fat proliferation in the subserosa. Sigmoid colon: ulceration, severe inflammation with surface inflammatory granulation tissue with vascular proliferation, thickened muscularis propria, vascular and mild fat proliferation in the subserosa.

A 68-year-old man had been diagnosed with left-sided UC 1 year and 7 months previously and had relapsed repeatedly despite treatment with pH-dependent 5-aminosalicylic acid (5-ASA) and topical budesonide. Two months before admission to our institution, his symptoms worsened, resulting in urgent admission. Remission induction therapy with prednisolone (PSL) 50 mg/day was started, but his disease was refractory to this treatment. Vedolizumab was introduced on Day 19 of hospitalization. Despite these treatments, the inflammation persisted, and IUS on Day 33 showed active inflammation in the left-sided colon (Figure 1). The D/C showed the most severe inflammation with mLS 4 and uncertain BWS, ulcers, and i-fat. In contrast, we found no evidence of inflammation in the A/C and T/C. His ongoing insufficient treatment response prompted laparoscopic subtotal colorectal resection on Day 38.

Histopathological examination of the operative specimen demonstrated severe inflammation, vascular proliferation, and thickened muscularis propria in the D/C and S/C, with deep ulceration in the D/C. The T/C showed submucosal fibrosis and muscularis propria thickening without active inflammation, whereas the A/C showed only mild submucosal fibrosis (Figure 1).

### Case 2

**Figure 2:**
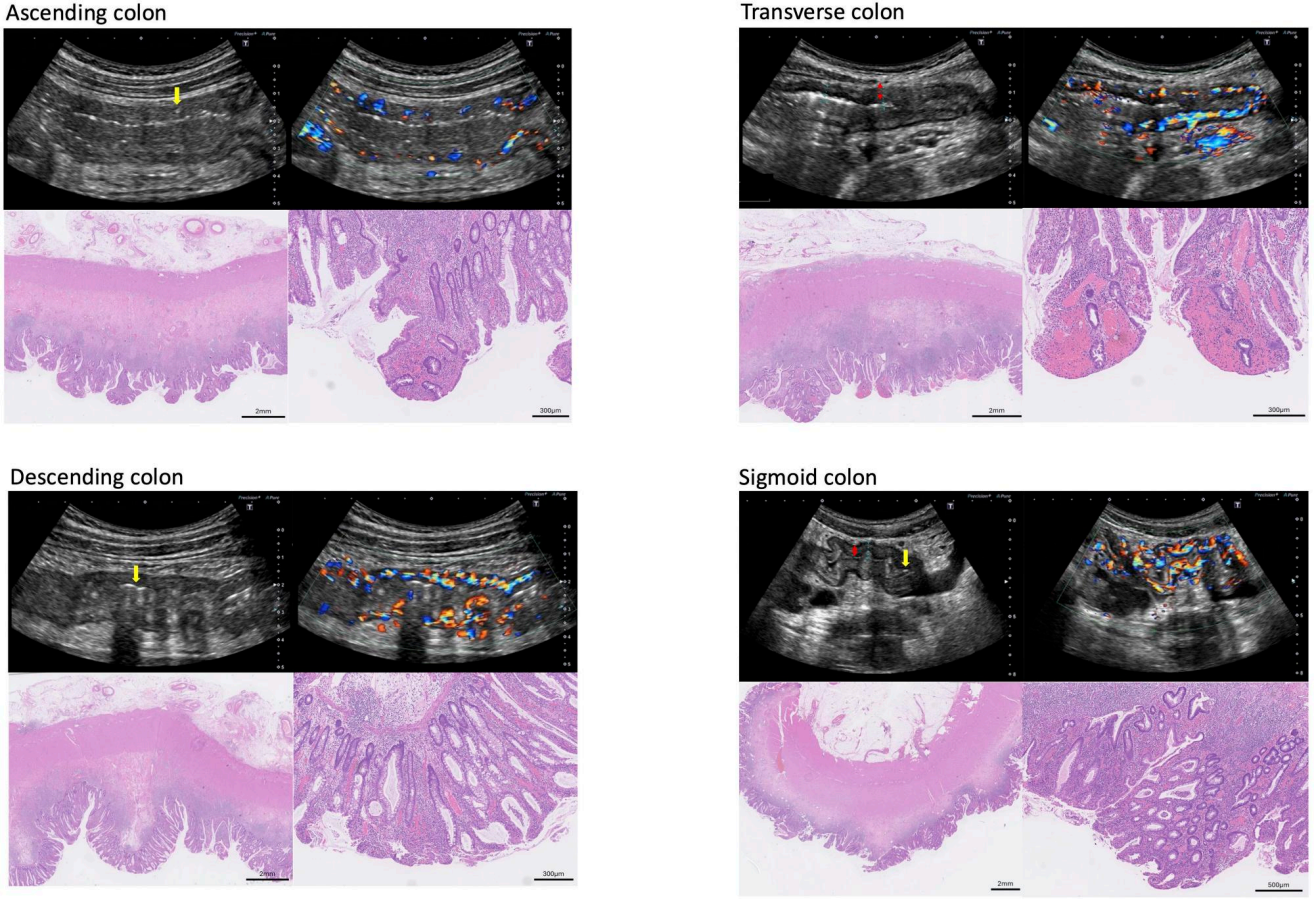
Transabdominal intestinal ultrasound images and histopathological findings in Case 2. Sonographic findings: Ascending colon: thickened bowel wall, loss of stratification, modified Limberg score 3, ulcers (+) (single arrow), i-fat (+). Transverse colon: thickened bowel wall, loss of stratification (double arrow), modified Limberg score 4, ulcers (+), i-fat (+). Descending colon: thickened bowel wall, loss of stratification, modified Limberg score 4, ulcers (+) (single arrow), i-fat (+). Sigmoid colon: thickened bowel wall, unclear stratification (double arrow), modified Limberg score 4, ulcers (+) (single arrow), i-fat (+). Histopathological findings (H&E staining): Ascending colon: erosion, severe inflammation in the mucosa and submucosa, fibrosis with vascular proliferation of the submucosal layer, thickened muscularis propria and fat tissue around the colon, vascular proliferation in mesenteric fat. Transverse colon: erosion and surface bleeding, severe inflammation in the mucosa and submucosa, edematous change with mild fibrosis and vascular proliferation of the submucosal layer, thickened muscularis propria. Descending colon: erosion, severe inflammation with vascular proliferation of mucosa and submucosa, edematous change with mild fibrosis of the submucosa, thickened muscularis propria, vascular proliferation in mesenteric fat. Sigmoid colon: ulceration, severe inflammation with surface inflammatory granulation tissue (vascular proliferation), edematous change with mild fibrosis of the submucosa, thickened muscularis propria.

A 16-year-old girl with a one-month history of bloody stools, diarrhea, and abdominal pain was diagnosed with pancolitis type UC by sigmoidoscopy. She was initially treated with time-dependent 5-ASA and PSL but her disease proved to be steroid-refractory. Accordingly, adalimumab and granulocyte/monocyte adsorption (GMA) were started at another hospital; however, her anemia and hypoalbuminemia worsened. Her treatment was switched to infliximab (IFX), after which she was transferred to our hospital. Despite treatment with IFX, followed by cyclosporine A and vedolizumab, disease control remained poor. IUS revealed severe BWT with loss of BWS and ulceration, particularly in the T/C, D/C, and S/C (mLS 4) (Figure 2). Given her persistent bleeding and IUS findings, a subtotal colorectal resection was performed on Day 16 of her hospitalization in our institution.

Histopathological examination of the operative specimen revealed severe inflammation with ulceration, granulation tissue, and thickened muscularis propria in all affected segments (Figure 2). The submucosa exhibited edema, mild fibrosis, and vascular proliferation. Mesenteric fat was prominent, particularly in the A/C and D/C.

### Case 3

**Figure 3:**
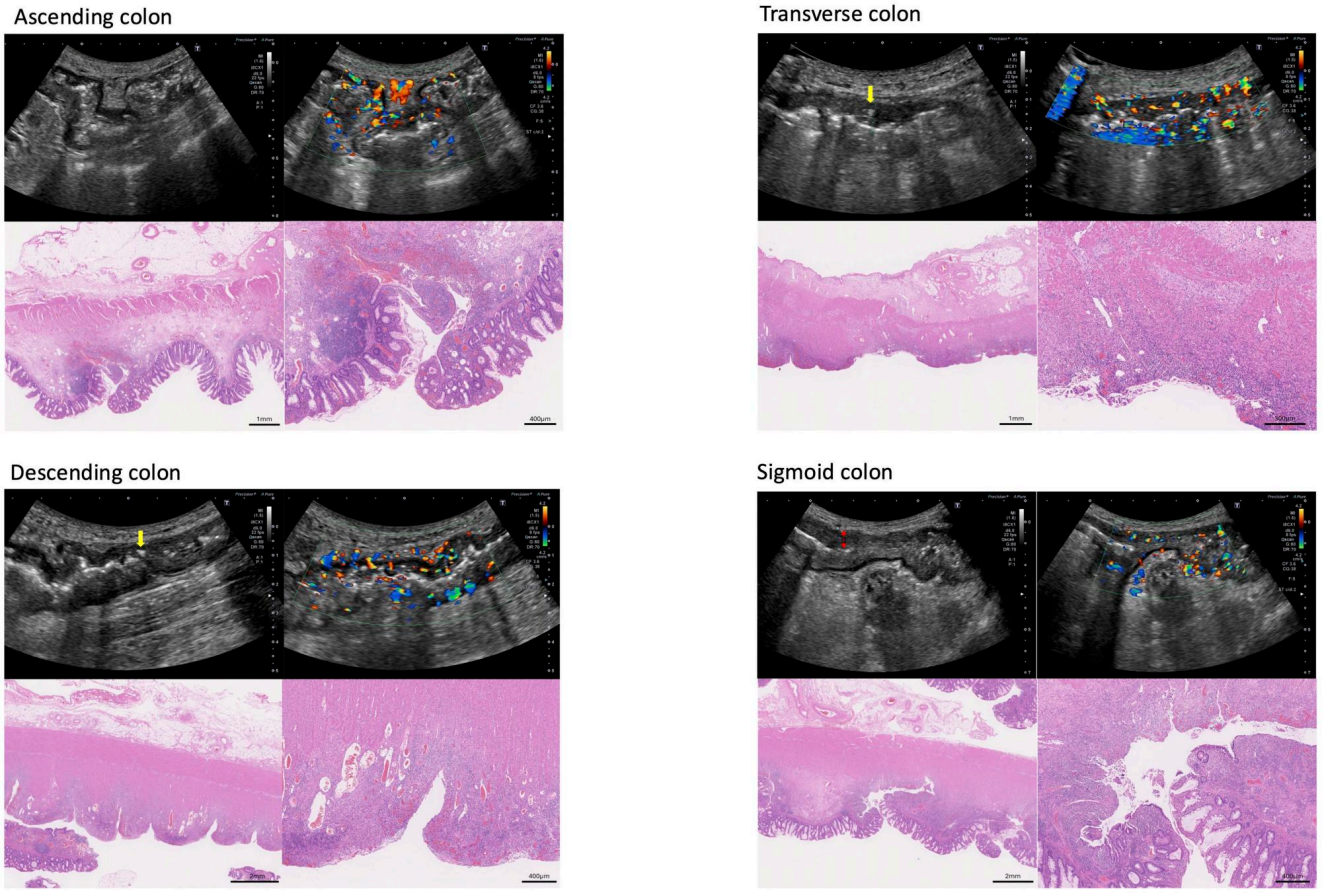
Transabdominal intestinal ultrasound images and histopathological findings in Case 3. Sonographic findings: Ascending colon: thickened bowel wall, loss of stratification, modified Limberg score 4, ulcers (+), i-fat (+). Transverse colon: thickened bowel wall, loss of stratification, modified Limberg score 4, ulcers (+) (single arrow), i-fat (+). Descending colon: thickened bowel wall, loss of stratification, modified Limberg score 4, ulcers (+) ( single arrow), i-fat (+). Sigmoid colon: thickened bowel wall, loss of stratification (double arrow), modified Limberg score 3, ulcers (+), i-fat (+). Histopathological findings (H&E staining): Ascending colon: ulceration, moderate to severe inflammation with surface inflammatory granulation tissue (vascular proliferation and bleeding), edematous change with mild fibrosis of the submucosa layer, fat tissue proliferation, and focal vascular proliferation in the subserosa. Transverse colon: deep ulceration, surface inflammatory granulation tissue (vascular proliferation) with severe inflammation, thickening of the subserosa with edematous change accompanied by mild inflammation and vascular proliferation. Descending colon: deep ulceration, surface inflammatory granulation tissue (vascular proliferation) with severe inflammation, thickened muscularis propria, fat tissue around the colon, and vascular proliferation in the subserosa. Sigmoid colon: ulceration, severe inflammation, and surface inflammatory granulation tissue (vascular proliferation), edematous change with fibrosis of the submucosa, thickened muscularis propria, vascular proliferation in mesenteric fat.

A 78-year-old woman had been diagnosed with UC 8 years previously and was on remission maintenance therapy with 2400 mg of MMX-type 5-ASA and probiotics. However, she developed abdominal pain and, 4 days later, bloody stools and increased abdominal pain. A further 3 days later, she was admitted to our hospital. She was diagnosed as having a relapse of UC. Accordingly, IFX was administered on Day 1 and again on Day 7. IUS performed at the time of the second dose of IFX showed severe active inflammation (Figure 3), thickened BWT, and loss of BWS from the T/C to S/C. Bowel wall vascularity was assessed as mLS 4 in the T/C and D/C and mLS 3 in the S/C. All segments exhibited ulceration and the presence of i-fat. Moreover, in the A/C, the colon wall appeared thin and there were multiple deep ulcers with mLS 4; dilatation of the bowel was also observed. Considering the severe active inflammation and risk of perforation and toxic megacolon and the lack of obvious clinical improvement, a laparoscopic total colectomy was performed on Day 10. A perforation of the T/C that had not been detected preoperatively was identified intraoperatively.

Histopathological examination of the operative specimen revealed severe inflammation with ulceration throughout the colon (Figure 3). Deep-seated ulcers with surface inflammatory granulation tissue were noted, particularly in the A/C and T/C. The submucosal layers of the A/C and S/C showed overall edema and mild fibrosis. Additionally, thickening of the muscularis propria was evident, particularly in the D/C and S/C. There was increased vascularization and proliferation of adipose tissue around the intestinal tract in all segments. Fat proliferation was remarkable, particularly in the subserosal adipose tissue and mesenteric fat in the A/C to D/C.

### Case 4

**Figure 4:**
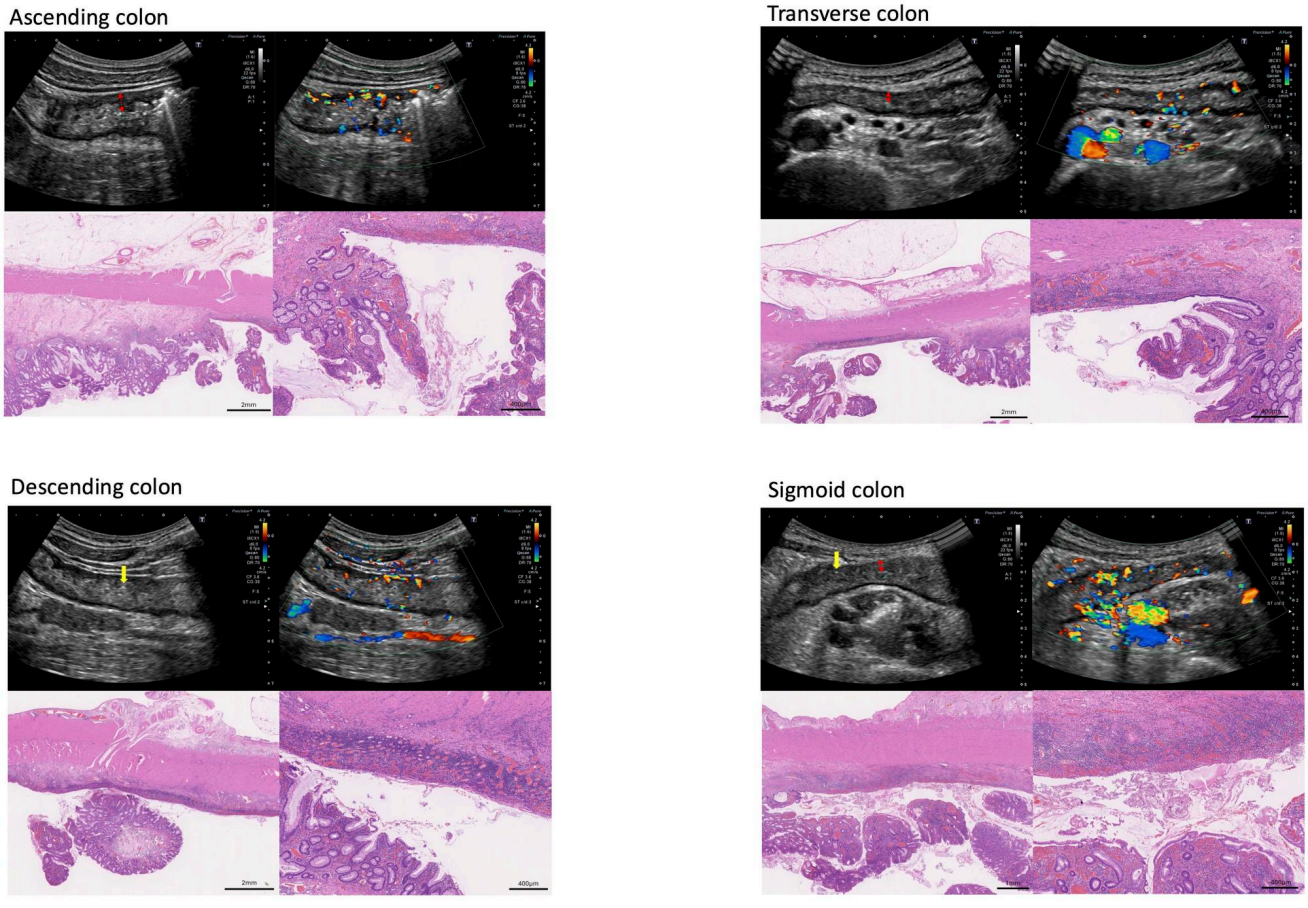
Transabdominal intestinal ultrasound images and histopathological findings in Case 4. Sonographic findings: Ascending colon: thickened bowel wall, unclear stratification (double arrow), modified Limberg score 3, ulcers (+), i-fat (+). Transverse colon: thickened bowel wall, unclear stratification (double arrow), modified Limberg score 3, ulcers (+), i-fat (+). Descending colon: thickened bowel wall, unclear stratification, modified Limberg score 3, ulcers (+) (single arrow), i-fat (+). Sigmoid colon: thickened bowel wall, loss of stratification (double arrow), modified Limberg score 3, ulcers (+) (single arrow), i-fat (+). Histopathological findings (H&E staining): Ascending colon: ulceration, severe inflammation with surface inflammatory granulation tissue (vascular proliferation), edema in the submucosal layer, mild thickening of the muscularis propria, fat proliferation in the subserosa. Transverse colon: ulceration, moderate to severe inflammation with surface inflammatory granulation tissue (vascular proliferation), thickened muscularis propria, vascular proliferation, and mild inflammation in the subserosa. Descending colon: ulceration, severe inflammation with surface inflammatory granulation tissue (vascular proliferation), thickened muscularis propria, vascular proliferation, and mild inflammation in the subserosa. Sigmoid colon: ulceration, severe inflammation with surface inflammatory granulation tissue (vascular proliferation), thickened muscularis propria, vascular proliferation with mild inflammation, and fibrosis in subserosa.

A 29-year-old man with pan-colitis type UC was transferred to our hospital after having been hospitalized in another institution for 2 months. On admission to the previous hospital, MMX 5-ASA and PSL 40 mg/day had been started. When the dose of PSL was reduced to 30 mg, his symptoms had worsened, in response to which the PSL dose was increased to 50 mg and granulocyte/monocyte adsorption and immunomodulators were added. Despite these changes, CS showed MES 3 without any improvement. After transfer, given that his disease was refractory to steroids, IFX was administered. After 2 weeks of IFX treatment, the Lichtiger index was 4 and C-reactive protein concentration was tending to improve, but was still above the normal limit. On IUS, all segments of the colon showed thickened BWT with loss of BWS, mLS 3, ulceration, and i-fat (Figure 4). The night after consuming food, the patient reported severe abdominal pain and was found to have rebound tenderness. A computed tomography scan showed free air, resulting in a diagnosis of gastrointestinal perforation, and emergency laparoscopic total colectomy was performed.

Histopathological examination of the surgical specimen showed moderate to severe inflammation with surface inflammatory granulation tissue and ulceration in all colonic segments (Figure 4). Thickening of the muscularis propria was present throughout all segments, being particularly remarkable in the D/C. Submucosal edema accompanied by mild fibrosis was prominent in the A/C. There was vascular proliferation in the subserosa throughout the colon, particularly in the D/C and S/C, where mild fibrosis was also found. Subserosal fat proliferation was characteristically observed in the A/C.

## Discussion

In the present study, thickened BWT and mLS 3-4 were commonly detected by transabdominal IUS in colonic segments found by histopathological assessment to harbor severe or moderate to severe transmural inflammation. Additionally, vascular proliferation (14/14 segments), edema (7/14 segments), and thickened muscularis propria (13/14 segments) were detected by pathological examination of these colonic segments. While there were trends, BWT values did not show statistically significant associations with edema, thickened muscularis propria, or fibrosis. The small sample size may have contributed to the low statistical power. Previous studies have reported correlations between these IUS findings and endoscopic evidence of mucosal surface inflammation.^11^^,^^12^ In this study, we gained new insights into the ability of IUS to assess transmural inflammation. Severe or moderate to severe inflammation (9/9 segments), vascular proliferation (9/9 segments), thickened muscularis (8/9 segments), and edema (5/9 segments) were extremely common histopathological findings in the nine segments with loss of BWS on IUS. In ultrasound physics, acoustic impedance is defined as resistance to transmission of ultrasound waves and ultrasound examination reveals boundaries where acoustic impedance varies between tissues.^13^ This is the mechanism by which IUS identifies BWS in the normal bowel wall. Therefore, loss of BWS occurs when the acoustic impedance is the same in each layer (eg, mucosa, submucosa, and muscularis).^14^ Our findings support the notion that inflammatory cell infiltration, vascular proliferation, and edema result in uniformity of bowel wall impedance, evidenced as loss of BWS.

An extra-intestinal finding, the sonographic parameter of i-fat, is associated with intestinal inflammation.^15^ However, what histopathological changes in i-fat denote in patients with UC has not yet been determined. In the present study, i-fat was found in all inflamed segments. Histopathological assessment showed fat proliferation in the subserosa in a limited number of segments (5/14 segments; two of these five segments showed only mild fat proliferation), whereas vascular proliferation in the subserosa was found in most segments (10/14 segments). These observations support the contention that i-fat in patients with UC reflects spread of colonic inflammation to the serosal side rather than proliferation of adipose tissue. Further, fat wrapping in Crohn’s disease presents as thickening of the fatty tissue around the end artery on the mesenteric side.^16^ Additionally, this characteristic pathological finding manifests as a peri-intestinal hyper-echoic region under IUS, similar to i-fat in UC.^17^^,^^18^ Our findings call attention to the fact that the histopathological findings in peri-intestinal hyperechoic areas differ between UC and Crohn’s disease and alert physicians to the importance of distinguishing between i-fat and fat wrapping: these terms should be used correctly.

In the cases of severe UC presented in this study, CS was not performed prior to surgery due to the high risk associated with the procedure. While CS is the gold standard for disease assessment in UC, its indication should be carefully considered. In patients with severe UC, deep insertion of CS can carry significant risks and is therefore not recommended. A key advantage of IUS is that it enables the safe evaluation of the entire colon and assessment of treatment response, even in situations where CS is considered inadvisable or not feasible. In Case 1, IUS did not reveal any evidence of inflammation in the A/C and T/C; histopathological examination also did not demonstrate active inflammation or vascular proliferation. Meanwhile, histopathological examination identified thickened muscularis propria and fibrosis in the submucosal layer in these segments, whereas no sonographic findings (eg, submucosal prominence or submucosal thickening) suggestive of submucosal fibrosis were identified. Although the definition of submucosal prominence has not been clearly established, applying the criterion proposed in a previous report,^19^ the segments with submucosal fibrosis did not appear to exhibit submucosal prominence.

These finding suggests that IUS has limited ability to detect intestinal fibrosis that may have been caused by previous inflammation. However, in retrospect, the haustrations seemed less smooth and rounded than they did in the normal colon. Regarding this difficulty in sonographically evaluating fibrotic change in the colon, Zhu et al. reported that fibrosis can be assessed by elastography.^20^ However, given that the normal thickness in the colon is < 3 mm,^11^ elastography of the intestinal wall would require exceptional skill and experience, restricting its use in general clinical practice. Meanwhile, considering that in this study many areas of hypervascularization were detected in the inflamed segments and superb micro-vascular imaging is superior to color Doppler in detecting minimal blood flow,^21^ assessing UC inflammation with this modality may be developed in the future.

One limitation of this study is that all participants had severe active UC and we did not compare their findings with those of control cases (eg, UC in remission). However, in clinical practice it is not feasible to obtain colectomy specimens from a patient with UC in remission who also undergoes IUS immediately preoperatively. A prospective study of patients with UC-associated cancer could include performing IUS preoperatively on all participants with the aim of investigating the correlations between IUS and histopathologic findings among patients with varying activity of UC. Such a study would provide further insights into the optimal transmural monitoring strategy with transabdominal IUS for UC.

## Conclusion

This is the first report comparing IUS findings obtained before colectomy with histopathological findings in resected colon specimens from patients with severe UC. The study suggests that transabdominal IUS findings demonstrate a high level of concordance with histopathological features of transmural inflammation. These results support the notion that IUS can serve as a non-invasive modality for assessing transmural disease activity beyond the mucosa in UC.

## Data Availability

The data underlying this article will be shared by the corresponding author upon reasonable request.
